# Changes in hand function and health state utility after cubital tunnel release using the United Kingdom Hand Registry

**DOI:** 10.1177/17531934241275487

**Published:** 2024-09-13

**Authors:** Joris S. Teunissen, Timothy T. Griffiths, Brigitte E. P. A. van der Heijden, Ryckie G. Wade, Jennifer C. E. Lane, Steven E. R. Hovius, Grainne Bourke, Fadi Issa, Jeremy N. Rodrigues, Conrad J. Harrison

**Affiliations:** 1 Department of Plastic, Reconstructive and Hand Surgery, Radboud Institute of Health Research, Radboud University Medical Centre, Nijmegen, The Netherlands; 2 Nuffield Department of Surgical Sciences, University of Oxford, Oxford, UK; 3 Leeds Institute for Medical Research, University of Leeds, Leeds, UK; 4 Department of Plastic, Reconstructive and Hand Surgery, Leeds Teaching Hospitals Trust, Leeds, UK; 5 Department of Plastic, Reconstructive and Hand Surgery, Jeroen Bosch Hospital, ‘-Hertogenbosch, Netherlands; 6 Nuffield Department of Orthopaedics, Rheumatology and Musculoskeletal Sciences, University of Oxford, Oxford, UK; 7 Clinical Trials Unit, Warwick Medical School, Warwick, UK; 8 Department of Plastic, Reconstructive, and Hand Surgery, Stoke Mandeville Hospital, Aylesbury, UK

**Keywords:** Patient-reported outcome measures, cubital tunnel syndrome, surgical procedures, quality of life, patient evaluation measure, outcome measurement

## Abstract

This study aimed to analyse and contrast changes in health-related quality of life (HR-QoL) and hand symptoms in the first 6 months after surgical treatment for primary cubital tunnel syndrome. Data originated from the United Kingdom Hand Registry. HR-QoL was assessed using the generic EuroQol five-dimensional assessment tool (EQ-5D-5L) and hand symptoms using the Patient Evaluation Measure (PEM). In total, 281 patients were included in the statistical analysis. Cubital tunnel release resulted in clinically relevant relief of hand symptoms. However, no improvement in HR-QoL was detected by the EQ-5D-5L. As a result, current health economic models, such as those used by the National Institute for Health Care Excellence (NICE) in the UK, might conclude that cubital tunnel release is not cost-effective. This discrepancy requires exploration, and hand-specific preference-based measures might be needed for value-based healthcare in hand surgery.

**Level of evidence:** III

## Introduction

Cubital tunnel syndrome (CuTS) is the second most common compression neuropathy of the upper extremity in the United Kingdom (UK), with an estimated annual incidence of 44/100,000 persons (Latinovic et al., 2006). If symptoms are not adequately improved using non-surgical modalities, surgical decompression can be considered (Boone et al., 2015). There are multiple surgical treatment options available. Recent work suggests that in-situ release is the safest operation and equally as effective as other procedures, but this topic remains controversial (Burahee et al., 2021; Caliandro et al., 2016; Wade et al., 2020).

Patient-reported outcome measures (PROMs) are questionnaires that evaluate a patient’s health in generic or condition-specific terms and can be used to evaluate the effectiveness of treatments, including surgery. In CuTS, there are a large variety of PROMs used. Previous research has found an improvement in patient-reported symptoms and hand function after surgical decompression as measured by the Boston Carpal Tunnel Questionnaire (BCTQ) and Disabilities of the Arm, Shoulder, and Hand (DASH) questionnaires (Mendelaar et al., 2022; Townsend et al., 2023). However, few studies have evaluated the change in health-related quality of life (HR-QoL) after surgical treatment for CuTS (Caliandro et al., 2016).

HR-QoL itself can be assessed by quantifying the desirability of health states described by PROM responses for a given population (such as UK inhabitants). For example, having moderate symptoms of anxiety might be less desirable than having no mobility but more desirable than always having intense pain. This preference-based scoring is termed ‘health state utility’ (HSU) (Feeny, 2000; Lenert and Kaplan, 2000). It is possible that this may vary between countries, so different HSU value sets exist for different countries (van Hout et al., 2012). HSU data are important to facilitate health economic processes, such as cost-utility analyses (Robinson, 1993), which might also aid in determining the optimal treatment strategy for patients with CuTS.

Therefore, the aim of the present study was to evaluate the change in HSU in the first 6 months after surgical treatment for CuTS using the national hand registry of the UK. In addition, we evaluated the change in hand symptoms using the Patient Evaluation Measure (PEM).

## Methods

### Study design

This observational cohort study uses data from the United Kingdom Hand Registry (UKHR) database, a voluntary national registry for quality assurance of surgical treatment outcomes for hand and wrist conditions. The data were prospectively obtained and retrospectively analysed. Patients who agreed to participate in the registry were asked to complete PROMs preoperatively and at predefined timepoints (2 and 6 months) postoperatively. Originally, PROMs were completed and returned by post. In 2018, the registry was updated, and PROM data were collected by email. For patients without email, PROM responses could be captured using Short Message Services (SMS). Results were collated by a central administrator independent of the operating surgeons.

Each patient provided written consent before inclusion into the registry, and identifiable data were anonymized before release from the registry for analysis. This study was exempt from ethical approval by the University of Oxford Clinical Trials and Research Governance. The study is reported following the Reporting of studies Conducted using Observational Routinely-collected Data (RECORD) guidelines (Nicholls et al., 2015).

### Patients

All consecutive adult patients who participated in the registry between February 2012 and February 2020 and received cubital tunnel surgery were identified and evaluated for eligibility.

The exclusion criteria for this study were as follows: (1) patients with missing demographics at baseline; (2) patients who underwent cubital tunnel decompression as part of revision surgery; (3) patients who did not complete PROMs at baseline and at least once after surgery; and (4) surgical variants for which less than 30 patients existed in the database (i.e. cubital tunnel decompression with submuscular transposition and/or medial epicondylectomy).

### Intervention

All patients underwent cubital tunnel surgery as chosen in conjunction with their operating surgeon. Operative details were uploaded to the UKHR online platform (https://www.ukhr.net). This study only evaluated in-situ cubital tunnel decompression and decompression with subcutaneous transposition.

### PROMs

Two PROMs are captured in the UKHR: the five-level EuroQol five-dimensional descriptive system (EQ-5D-5L) and the PEM. Patients undergoing cubital tunnel surgery were asked to complete both PROMs at intake and at 2 months and 6 months postoperatively.

The EQ-5D-5L is a generic health status measure evaluating five dimensions of health (1: mobility; 2: self-care; 3: usual activities; 4: pain/discomfort; 5: anxiety/depression) representing the global HR-QoL (EuroQol–a new facility for the measurement of health-related quality of life, 1990; Rabin and de Charro, 2001). The preference-based scoring of the EQ-5D-5L for the UK, i.e. the UK utility index set, ranges from −0.594 to 1.0, where 1 reflects the best health state utility imaginable, 0 is ‘death’ and negative values are considered worse than death (van Hout et al., 2012).

The PEM measures hand function (Dias et al., 2008, 2001; Macey et al., 1995). Between 2012 and 2017, the UKHR captured the original 10-item version of the PEM (Macey et al., 1995). This was changed to the updated 11-item version of the PEM in 2017 (Dias et al., 2001), which has an additional question concerning the duration of pain. As this item was missing for most of the patients in the registry, we chose to use complete response sets of the original 10-item for the analysis. Our analysis did not include parts 1 or 3 of the PEM questionnaire as these parts measure the care process and are more akin to a patient-reported experience measure, not a hand function measure.

### Data-access and cleaning methods

We had access to participants’ demographics (sex, age), surgical procedure and item-level data for both PROMs at each timepoint. The EQ-5D-5L utility index was calculated using the UK value set for each timepoint using the EQ-5D-3L crosswalk value (van Hout et al., 2012). The PEM total score was calculated as the sum of the item response scores (range of possible scores 10–70; lower scores indicate better hand performance).

### Study size and statistical analyses

The study size was determined by convenience, as the number of eligible patients added to the registry between February 2012 and July 2020. We performed a linear mixed-effects model (LMM) for repeated measures to estimate the change in PROM scores between intake and each postoperative timepoint. Two LMMs were made: one for the EQ-5D-5L and one for the PEM. The fixed effects of the model were timepoint, age and sex, while the random effect was the individual patient. The estimated marginal mean, including a 95% confidence interval (CI), was computed for each timepoint and compared with Tukey’s adjustment for multiple testing. Scores were calculated for all patients as one group and stratified based on the surgical procedure. The outcomes of different surgical procedures were not compared, because we had no access to data that informed why one surgical approach had been chosen over another. Missing data (approximately 15%) were not imputed as they do not have added value in LMM (Peters et al., 2012). A *p*-value <0.05 was considered statistically significant. Baseline characteristics of patients included in the EQ-5D-5L and PEM analysis were compared using effect sizes and the overlap between the cohorts was calculated. Lastly, a sensitivity analysis was performed in which the LMMs were repeated with only the subgroup of patients who completed both the PEM and EQ-5D-5L.

### Assessment of differences between completers and non-completers

As participation in the BSSH registry is voluntary, missing data were expected (Hutchings et al., 2012). To evaluate the potential risk of selection bias from loss to follow-up, the cohort of patients who completed PROMS after surgery (completers [C]) and patients who did not (non-completers [NC]) were compared at baseline using Cohen’s D effect sizes for numeric data and Cliff’s delta for categorical data (Cohen, 1992).

### Estimating the MIC for the PEM in this patient cohort

We attempted to estimate the minimal important change (MIC) for the PEM to evaluate whether the observed change was also clinically relevant. We calculated the MIC as half the standard deviation (SD) of the PEM at baseline (Norman et al., 2003), which is commonly used in clinical trials.

## Results

**Figure 1.**
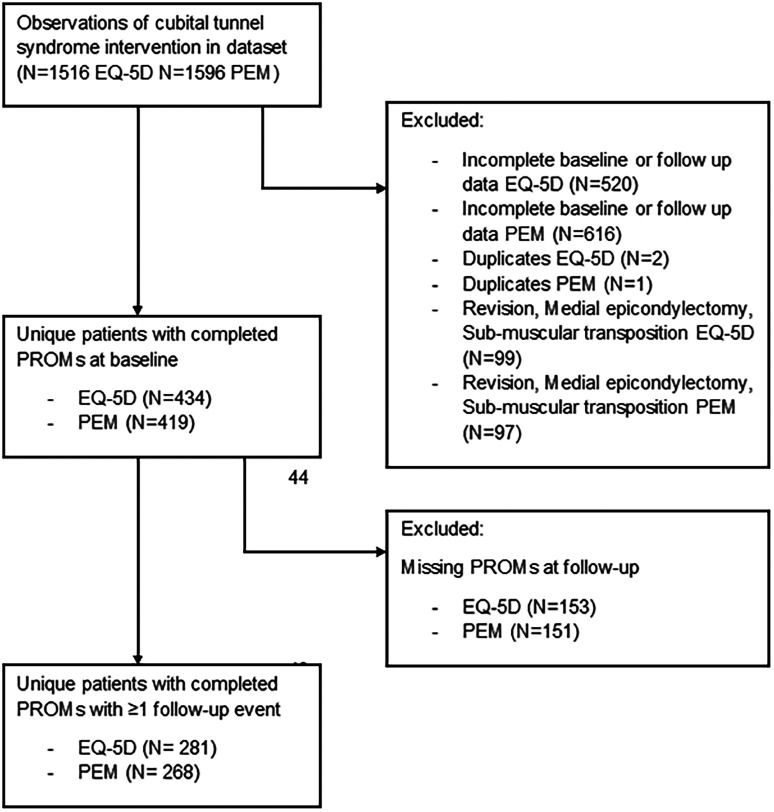
Flow chart of the study.

Between February 2012 and July 2020, 565 unique patients with CuTS were entered into the registry. Of those patients, 281 (50%) fulfilled the inclusion criteria for the analysis of EQ-5D-5L scores and 268 (47%) for the analysis of PEM scores. There was an 82% overlap in patients between the two analyses. The reasons for exclusion are summarized in Figure 1. Retention rates at 6 months after surgery were high, in the range of 74%–83%; the number of patients analysed at each timepoint is shown in Table 1.

**Table 1.** Overview of data availability of the patients included in the linear mixed models.

	PEM			EQ-5D-5L		
	All	ISD	ST	All	ISD	ST
Intake (*n*)	268	222	46	281	225	56
2 months (*n*)	208	174	34	216	170	46
6 months (*n*)	204	166	38	214	171	43
Retention rate (%)^a^	76	74	83	76	76	77

a Calculated as N_6 months_/N_Intake_ × 100.

EQ-5D-5L: EuroQol five-dimensional assessment tool; ISD: in-situ decompression; PEM: Patient Evaluation Measure; ST: subcutaneous transposition.

The demographics of patients included in the EQ-5D-5L and PEM analyses are shown in Table 2, which confirms that patients in both groups were similar (Cohen’s |d| < 0.2 ‘negligible differences’). In addition, the included patients (C) were similar in sex, operation type and baseline scores to patients who were not included (NC), but somewhat older (Table S1).

**Table 2.** Summary characteristics of the patients included in the linear mixed models of the PEM and EQ-5D utility index.^a^

Characteristic	PEM	EQ-5D-5L	Effect size^b^
No. of patients	268	281	–
Age (years)	55 (45–65)	57 (47–69)	0.19
Sex (F)	134 (50)	140 (50)	0.00
*Operation*			0.03
In-situ decompression	222 (83)	225 (80)	–
Subcutaneous transposition	46 (17)	56 (20)	–

Data are *n* (%) or median (IQR) unless otherwise indicated.

a The overlap in patients between the two analysis is 82%.

b Cohen’s D for numeric variables and Cliff’s delta for categorical variables. The magnitude was interpreted as Cohen’s |d| < 0.2: negligible, |d| < 0.5: small, |d| < 0.8: medium, otherwise large (Cohen, 1992) and Cliff’s |d| < 0.147: negligible, |d| < 0.33: small, |d| < 0.474: medium, otherwise large.

EQ-5D-5L: EuroQol five-dimensional assessment tool; IQR: interquartile range; PEM: Patient Evaluation Measure.

**Figure 2.**
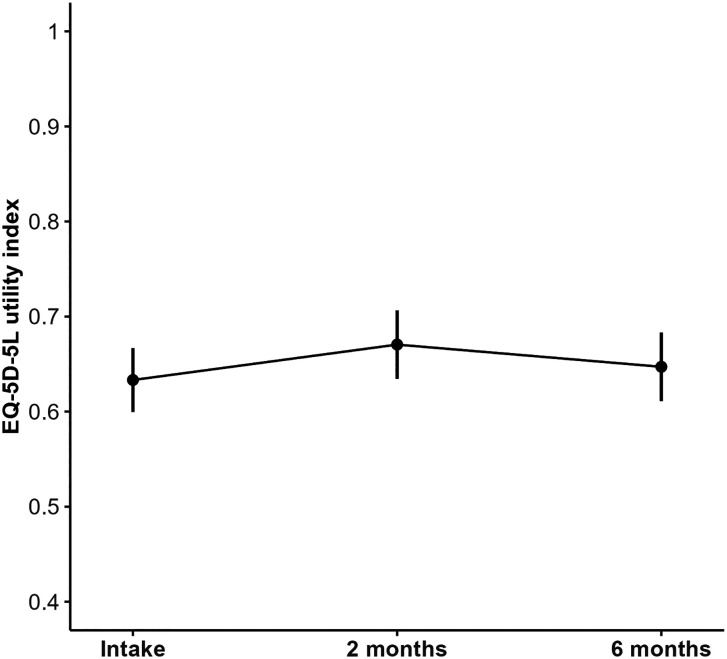
Adjusted marginalized means (with 95% confidence intervals) of the EQ-5D-5L utility index before cubital tunnel release and at 2 and 6 months postoperatively.

The mean EQ-5D utility index did not show significant improvement with a value of 0.63 (95% CI: 0.60 to 0.67) at intake, 0.67 (95% CI: 0.63 to 0.68) at 2 months and 0.65 (95% CI: 0.61 to 0.68) at 6 months (*p* = 0.99) (Figure 2). Mean EQ-5D-5L utility index scores were similar for the two types of operation (Table 3).

**Table 3.** Adjusted marginalized means (with 95% CIs) derived from the linear mixed models.

PROM	Intake	2 months	6 months
*PEM*			
All	41 (39 to 42)	29 (27 to 31)^b^	30 (28 to 32)^b^
In-situ decompression	41 (39 to 42)	29 (27 to 31)^b^	29 (27 to 31)^b^
Subcutaneous transposition	41 (37 to 45)	30 (25 to 34)^b^	33 (29 to 37)^b^
*EQ-5D utility index*			
All	0.63 (0.60 to 0.67)	0.67 (0.63 to 0.68)	0.65 (0.61 to 0.68)
In-situ decompression	0.62 (0.59 to 0.66)	0.66 (0.62 to 0.70)	0.64 (0.60 to 0.68)
Subcutaneous transposition	0.67 (0.61 to 0.74)	0.70 (0.63 to 0.77)	0.68 (0.61 to 0.75)

Comparison to intake value ^a^*p* < 0.05, ^b^*p* < 0.001.

CI: confidence interval; EQ-5D-5L: EuroQol five-dimensional assessment tool; PEM: Patient Evaluation Measure.

**Figure 3.**
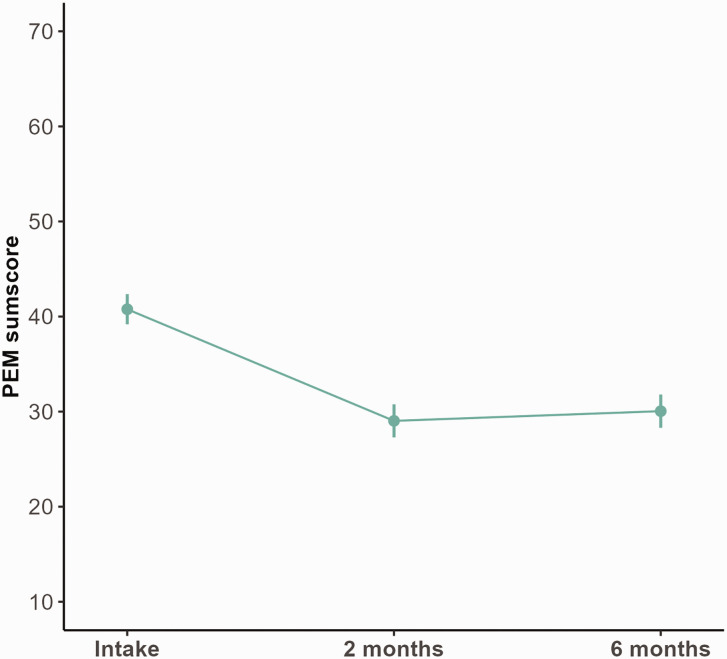
Adjusted marginalized means (with 95% confidence intervals) of the PEM score before cubital tunnel release and at 2 and 6 months postoperatively.

The mean PEM score improved from 41 (95% CI: 39 to 42) at intake to 29 (95% CI: 27 to 31) at 2 months (*p* < 0.001) (Figure 3; Table 3). This improvement was larger than 0.5 SD at intake (6.5), indicating a clinically relevant change. At the 6-month follow-up, the mean PEM score was 30 (95% CI: 28 to 32), which was not significantly different from the 2-month follow-up (*p* = 0.99). Improvements in PEM scores were seen for both types of surgery (Table 3). The sensitivity analyses on the 231 patients who filled in both PROMs showed similar estimations for the PEM and EQ-5D-5L (Table S2), indicating the robustness of the results.

## Discussion

This study shows that for patients with CuTS, in-situ decompression with or without subcutaneous transposition demonstrates a clinically relevant improvement in hand symptoms (as measured with the PEM) at 2 months postoperatively, which remains at 6 months. However, improvements in hand symptoms were not paralleled in generic health state utility (as measured by the EQ-5D-5L). There are two possible explanations for this important discrepancy. It may be that the EQ-5D-5L is not sensitive to meaningful changes in hand function after treatment. Alternatively, the improvement in hand function demonstrated by the PEM change is not perceived to be of high value to the UK population.

Currently, evaluation of changes in HR-QoL and health economic analyses in hand surgery rely on generic preference-based measures (such as the EQ-5D-5L) as no hand-specific preference-based measure exists. Because the EQ-5D-5L is not specific to hand conditions, it might not be able to capture important impacts of hand function on the quality of life. For example, the EQ-5D-5L asks about washing and dressing, usual activities, pain, anxiety and general mobility, but not about elements of health for which the nerve decompression is performed such as pain, weakness or paraesthesia. This means that surgical treatments for hand conditions are at risk of being undervalued when assessed with the EQ-5D-5L for cost-effectiveness analyses. Consequently, treatments might be unfairly labelled as being cost-ineffective or of ‘limited clinical value’ (NICE, 2019; Shewring et al., 2018). However, as stated, it may be that the hand function changes are not perceived as important to the general population, and this needs to be considered rationally as well. The discrepancy we have described here requires further exploration and possibly the development of hand-specific preference-based measures for economic health evaluation in hand surgery. This has already been successfully undertaken in the field of breast reconstruction, for similar reasons (Kaur et al., 2021).

Patients with CuTS reported an improvement in hand function at 2 months postoperatively when completing the PEM. The improvement in PEM score was observed for both in-situ decompression and those undergoing subcutaneous transposition, with wider confidence intervals for decompression with subcutaneous transposition owing to a smaller sample size. Improvement in patient-reported symptoms after cubital tunnel surgery seems consistent throughout the literature irrespective of the hand-specific PROM used (Mendelaar et al., 2022; Townsend et al., 2023). Therefore, our results on the improvement of hand symptoms after surgery are in line with previous research.

This study has some limitations. First, participation in the registry is currently voluntary, and only a fraction of the patients who undergo cubital tunnel surgery in the UK are entered into the dataset annually. In addition, 26% of the patients who provided preoperative data were lost during follow-up. Therefore, it is uncertain to what extent the results from this study are generalizable to the UK population. To increase participation in and adherence to the registry, administrative burdens for clinicians and patients should be minimized. Therefore, we have recently developed a computerized adaptive test version of the PEM for patients with CuTS and thumb base osteoarthritis to reduce the questionnaire length by 80% (Kamran et al., 2022; Teunissen et al., 2023). Implementation of these reduced questionnaires may boost response rates and improve the generalizability of the UKHR.

Second, the minimal important change (MIC) of the PEM in patients with CuTS had not been reported before. Therefore, we tried to estimate the MIC with a rule of thumb that is commonly used in clinical trials and states that the threshold of discrimination for changes in HR-QoL is consistently approximately half the SD at baseline (Norman et al., 2003). A future study should calculate the MIC for the PEM for all common hand conditions using an anchor-based approach to better interpret the clinical meaning of statistical changes.

Third, we were unable to investigate which surgical treatment options yield the best outcomes for patients with cubital syndrome. Some operation options (e.g. medial epicondylectomy) were excluded from analyses due to low sample sizes that would lead to inadequate statistical power. Furthermore, the registry does not capture any information on the clinical decision-making to perform one surgery over the other. For example, we did not know whether the treating surgeon always performed transposition or if it was only based on clinically evident subluxation. To make meaningful comparisons between treatment options using the UKHR, more clinical data need to be captured.

This study adds to the evidence that valid, responsive, consistent, disease-specific measures should be included in any considerations by funding bodies alongside generic health measures (Patrick and Deyo, 1989). Future research will focus on the development of a hand-specific preference-based measure that can detect meaningful changes in hand function after treatment to allow health economic evaluations.
